# Peroxisome proliferator-activated receptor agonists modulate neuropathic pain: a link to chemokines?

**DOI:** 10.3389/fncel.2014.00238

**Published:** 2014-08-20

**Authors:** Caroline M. Freitag, Richard J. Miller

**Affiliations:** Department of Molecular Pharmacology and Biological Chemistry, Richard J. Miller Laboratory, Northwestern UniversityChicago, IL, USA

**Keywords:** neuropathic pain, MCP-1, RANTES, MIP-1α, fractalkine, SDF-1, peroxisome proliferator-activated receptors

## Abstract

Chronic pain presents a widespread and intractable medical problem. While numerous pharmaceuticals are used to treat chronic pain, drugs that are safe for extended use and highly effective at treating the most severe pain do not yet exist. Chronic pain resulting from nervous system injury (neuropathic pain) is common in conditions ranging from multiple sclerosis to HIV-1 infection to type II diabetes. Inflammation caused by neuropathy is believed to contribute to the generation and maintenance of neuropathic pain. Chemokines are key inflammatory mediators, several of which (MCP-1, RANTES, MIP-1α, fractalkine, SDF-1 among others) have been linked to chronic, neuropathic pain in both human conditions and animal models. The important roles chemokines play in inflammation and pain make them an attractive therapeutic target. Peroxisome proliferator-activated receptors (PPARs) are a family of nuclear receptors known for their roles in metabolism. Recent research has revealed that PPARs also play a role in inflammatory gene repression. PPAR agonists have wide-ranging effects including inhibition of chemokine expression and pain behavior reduction in animal models. Experimental evidence suggests a connection between the pain ameliorating effects of PPAR agonists and suppression of inflammatory gene expression, including chemokines. In early clinical research, one PPARα agonist, palmitoylethanolamide (PEA), shows promise in relieving chronic pain. If this link can be better established, PPAR agonists may represent a new drug therapy for neuropathic pain.

## Introduction

Chronic pain presents a serious medical problem. Current pain therapies show limited efficacy and many patients experience pain that is refractory to the available treatments. Neuropathic pain is frequently characterized by inflammation which can lead to sensitization in both the central and peripheral nervous systems. Key inflammatory mediators that are known to participate in chronic pain, including chemokines, have emerged as new therapeutic targets. Here, for the first time, we present a review of the literature linking chemokines in neuropathic pain to activation of peroxisome proliferator-activated receptors (PPARs). Ligand bound PPARs are known to inhibit the expression of inflammatory genes by a process termed *transrepression*. Among the genes repressed by activated PPARs are those of chemokines and their receptors. Early clinical trials indicate that PPAR agonists can be effective at alleviating neuropathic pain, even in patients who failed to respond to other treatments. While much remains to be understood about how PPAR agonists achieve this effect, it seems probable that inhibiting the expression of pain-causing inflammatory mediators like chemokines represents at least one mechanism for pain reduction.

## Neuropathic pain

Pain is defined as an unpleasant sensation induced by a noxious stimulus. There are two commonly used criteria for distinguishing acute from chronic pain. Acute pain is typically defined as pain associated with an injury and pain that is relatively short in duration. Chronic pain is sometimes defined as pain that persists beyond the expected healing time of an injury. Alternatively, researchers and clinicians may use arbitrary time points to define chronic pain as pain that persists beyond this time frame, e.g., 3 months. Acute pain serves an important function by warning individuals of tissue damage. Chronic pain, when it is dissociated from an injury, does not serve this purpose. Instead, chronic pain results from dysregulation, also called sensitization, of the nervous system. Persistent pain can produce permanent functional changes in the pain perception pathway. Sensitization can occur at all levels of the pain neuraxis, in both the central and peripheral nervous systems (Costigan et al., 2009).

Chronic pain can be divided into two classes, nociceptive and neuropathic. Nociceptive pain is caused by activation of nociceptors in the skin, tissue, or viscera in response to injury. Neuropathic pain results from damage to the somatosensory nervous system. Peripheral neuropathies may involve injured sensory, motor, or autonomic nerves. In the central nervous system, injury, stroke, or disease in the brain or spinal cord can also generate a state of chronic, neuropathic pain. These causes of neuropathic pain often evoke a strong immune response (Woolf and Mannion, 1999; von Hehn et al., 2012).

### Inflammation

Animal models of neuropathic pain have illuminated some of the complex mechanisms that underlie the development and maintenance of pain states after injury. Researchers have been able to reproduce human-like pain responses in animals, and study the mechanisms that generate such pain behaviors as well as possible treatments. Neuropathic pain symptoms are often heterogeneous in nature, and animal models have shown that several mechanisms are likely involved. Mechanisms including neuronal hyperexcitability (Wall and Gutnick, 1974; Empl et al., 2001; Wu et al., 2002; Coull et al., 2005; Jung et al., 2008; Bedi et al., 2010), changes in gene expression (Plunkett et al., 2001; Barclay et al., 2002; Bhangoo et al., 2007; Sandhir et al., 2011), and alterations in the neuronal environment (Frisén et al., 1993; Sommer et al., 1993; Zelenka et al., 2005) not only contribute to neuropathic pain, but may also facilitate and enhance one another. Physical damage to the nervous system, as well as changes in chemical and electrical signals in and around neurons contributes to pain.

Inflammation is an adaptive response to bodily insults like infection and tissue injury. The immune system response to nerve injury alters the chemical environment of sensory and pain neurons. Evidence points to a role for immune cells and inflammatory mediators in generating not only inflammatory pain but chronic, neuropathic pain as well (Moalem and Tracey, 2006; Medzhitov, 2008).

Many inflammatory mediators have been implicated in cases of neuropathic pain, yet to what degree immune system actions specifically cause and/or maintain neuropathic pain is incompletely understood. Research in animal models supports the conclusion that neuroimmune signaling contributes to sensory dysregulation and neuropathic pain. At the most fundamental level, injured neurons and glia release inflammatory mediators that activate resident and recruit circulating immune cells. These cells then release cytokines and chemokines that can alter neuronal signaling (Calvo et al. (2012) have written a superior review on this topic).

### Treatments

Recent epidemiological studies have placed the prevalence of chronic, neuropathic pain at 6–8% in the general population (Torrance et al., 2006; Bouhassira et al., 2008). However, the occurrence of pain differs greatly between neuropathies. For example, the prevalence of neuropathic pain in spinal cord injury patients is between 25–60%; while 70–90% of patients suffering from Guillain-Barré Syndrome report neuropathic pain (Moulin, 1998; Werhagen et al., 2004). Symptoms are many and vary from patient to patient. Pain phenotypes are not always specific to a neuropathy, and pain can result from neuropathy as well as from medications taken to treat the condition (Nandi, 2012). Patients may present multiple pain phenomena simultaneously, and their pain phenotypes can change over time. These observations suggest that different mechanisms may be at play within a particular neuropathic condition and even within a single patient.

Several groups of drugs have been utilized in neuropathic pain treatment; among them are analgesics like opiates, anti-inflammatory drugs including steroids, tricyclic antidepressants, anticonvulsants, antiepileptics, antihypertensives, local anesthetics, sodium channel blockers, NMDA receptor antagonists, SSRIs (selective serotonin-reuptake inhibitors), and cannabinoids (Moulin, 1998; Pöllmann and Feneberg, 2008; Park and Moon, 2010; Nandi, 2012). Side effects are common, and the use of nearly all these medications is complicated by concerns about their safety and efficacy. Apprehensions about drug dependence, tolerance, and other side effects arise when drugs are used chronically, especially at increasing doses. In some cases, patients may benefit from a treatment for a time, suddenly stop responding, and require a new therapy. For the most extreme neuropathic pain conditions, drugs may incompletely treat pain or fail to do so altogether (Harden and Cohen, 2003). Drugs that are well tolerated and effective at treating the most severe pain have yet to be developed.

## Chemokines

Mediators, such as cytokines and chemokines, are vital messengers in the inflammatory process playing roles as both proinflammatory and anti-inflammatory/prorepair signals that act upon numerous target tissues. Cytokines and chemokines are capable of directly influencing nociceptive transmission at every level of the pain neuraxis (Myers et al., 2006).

Chemokines (the name is derived from their function as CHEMOtactic cytoKINES) are small signaling molecules that serve as inflammatory mediators. Chemokine ligands are grouped into four families based on their amino acid sequence: alpha (CXC), beta (CC), gamma (C), and delta (CX3C). These designations refer to the positions of two conserved cysteine residues near the peptide’s n-terminus. Chemokines exert their functions by binding to a family of seven transmembrane g-protein coupled receptors (GPCRs), which are given names correlated to the ligands they bind.

Chemokines were first identified for their role in inflammation (Yoshimura et al., 1987). Chemokines are released by damaged cells and have a vital function in facilitating the migration of leukocytes to the lesioned area (Charo and Ransohoff, 2006; Savarin-Vuaillat and Ransohoff, 2007). However, researchers discovered that while diversification of chemokines and their receptors correlates with the development of a complex immune system, some chemokines predate the evolution of the immune system (Huising et al., 2003; DeVries et al., 2006). Specifically, SDF-1 (stromal cell derived factor 1; CXCL12) and its cognate receptor, CXCR4, are found in life forms without immune systems. Further, SDF-1 and CXCR4 are constitutively expressed when many chemokines are upregulated only during inflammation. This discovery prompted increased research into chemokines and their receptors. Now more than 50 chemokines and 20 receptors have been identified, and the known roles they play are more varied.

Chemokine signaling is important for immune system homeostasis (immune surveillance and immune cell maturation) as well as for inflammation. Chemokines also serve key functions in hematopoiesis, angiogenesis and neurodevelopment. Indeed, these roles are still observed in the adult, as SDF-1/CXCR4 signaling plays a role in adult neurogenesis (Lu et al., 2002) as well as generating tumor vasculature (Koshiba et al., 2000; Rempel et al., 2000). More recent research has also demonstrated that chemokines can be potent neuromodulators. They can regulate neurotransmitter release, alter ion channel activity, and even act as neurotransmitters themselves (Qin et al., 2005; White et al., 2005a; Zhang et al., 2005; Sun et al., 2006; Jung et al., 2008).

### Chemokine signaling in chronic inflammation and neuropathic pain

Chemokine expression is a downstream effect of the inflammatory cascade. Chemokine transcription is typically stimulated by “upstream cytokines” like interleukin-1β (IL-1β) and tumor necrosis factor-α (TNFα). The upregulation of IL-1β and TNFα by sensory neurons is a very early, post trauma event (Uçeyler et al., 2007; Sacerdote et al., 2008). Chemokines are capable of selectively recruiting monocytes, neutrophils, and lymphocytes, by establishing a chemical concentration gradient, or “chemokine gradient”. Cells expressing cognate chemokine receptors travel this gradient toward the location of highest chemokine concentration. Chemokines not only act on their receptors to make immediate alterations to cell signaling but also activate the expression of further downstream inflammatory mediators.

Chemokines are expressed both as part of the normal inflammatory response and as part of the pathology of chronic inflammation. Chemokine signaling has been implicated in conditions ranging from autoimmune disorders to vascular and pulmonary diseases, transplant rejection, and cancer. In neurological diseases with an inflammatory component, such as multiple sclerosis, Alzheimer’s disease and HIV-1 infection, research has shown that chemokines serve many key roles, including the generation and maintenance of disease associated neuropathic pain. Chemokine expression is also observed in many animal models of neuropathy induced pain.

Oh et al. (2001) made an important connection between chemokines and pain *in vivo* when they demonstrated that injection of SDF-1, RANTES, and MIP-1α could produce hindpaw tactile allodynia in rats. In neuroinflammation, chemokines are released not only by resident and recruited immune cells but also by damaged, inflamed nervous system cells. Further, neurons and glial cells that produce chemokines are also targeted by those same signals. DRG neurons in culture express chemokine receptors including CXCR4, CCR4, CCR5, and CX3CR1, the fractalkine receptor (Oh et al., 2001). Additionally, a subset of cultured DRG neurons demonstrated strong excitation in response to administration of chemokines including SDF-1, MCP-1, RANTES, and fractalkine (Oh et al., 2001; White et al., 2005b). Chemokines are coexpressed in neurons along with pain associated neurotransmitters including CGRP and substance P (Oh et al., 2001; Li et al., 2003; Dansereau et al., 2008). Excitation by chemokines, including CXCL1 and MCP-1, also prompt the release of CGRP, further strengthening the connection between chemokines and pain (Qin et al., 2005; Jung et al., 2008).

It is well known that chemokines and other proinflammatory mediators make a cytotoxic environment that strongly affects local cells (Frisén et al., 1993; Sommer et al., 1993). Further, chemokine upregulation can persist for weeks after injury in animal models (Flügel et al., 2001; Zhang and De Koninck, 2006; Bhangoo et al., 2007). Thus, persistent chemokine upregulation is not only consistent with a role in hypersensitizing nociceptors, but also provides an attractive therapeutic target.

### Targeting chemokine signaling to treat neuropathic pain

Several of the pain treatments described above, such as tricyclic antidepressants and NMDA receptor blockers, act primarily upon neuronal targets. As neuron-glial cell interactions have been recognized as fundamental to pain pathology, drugs that target messengers like cytokines and chemokines which signal between these different cells have drawn more attention. Several methods may be useful in disabling chemokine-receptor communication including antibodies and antagonists. Pharmaceutical companies have developed and tested antagonists to a number of cytokine and chemokine receptors with mixed results.

For example, CCR2 receptor antagonists (CCR2-RAs) are capable of temporarily relieving pain in some animal models when administered after the establishment of neuropathic pain. CCR2-RAs can block established pain for a matter of hours after injection in an lysophophatidylcholine (LPC) model (Bhangoo et al., 2007), a chronic constriction injury model (Serrano et al., 2010; Van Steenwinckel et al., 2011), a trigeminal pain model (Zhang et al., 2012), and a chemotherapy drug induced pain model (Pevida et al., 2013). A recent study by Padi et al. (2012) used a CCR2/CCR5 receptor antagonist to treat pain. They propose that a broad-spectrum chemokine receptor antagonist may be a more powerful therapy.

In spite of their promise, very little data has been published on the use of CCR2-RAs to treat pain in human neuropathy. Pease and Horuk (2009) describe CCR2-RAs in clinical trials for a variety of human disease conditions, not simply pain treatment (Pease and Horuk, 2009). Kalliomäki et al. (2013) published an inconclusive study using a novel CCR2-RA to treat post traumatic neuralgia, or pain following a traumatic event such as surgery, injection, and radiation. The study recruited test subjects with established pain and compared several pain measures taken before and after treatment. The researchers reported no significant improvement in pain symptoms on any measure between either drug group and placebo. However, they did show an increase in plasma MCP-1, and decreased monocyte levels suggesting that the antagonist had in fact acted upon its target. In the end the authors attributed their underwhelming results to tester variability, too many patient test centers, and a heterogeneous population of pain types and causes (Kalliomäki et al., 2013).

While antagonists are one important avenue of therapy, their limitations argue strongly for the development of drugs that can better block chemokine/receptor communication. A method for targeting chemokine signaling this way may be to limit the gene expression of the chemokine and/or receptor. As long-term changes in gene expression underlie the persistent upregulation of chemokines in chronic pain, changes in a gene’s transcriptional regulation may allow alterations of that gene’s expression level. Thus, in order to counteract the harmful chemokine upregulation seen in chronic pain, targeting the regulatory elements of transcription may be fruitful.

## Peroxisome proliferator-activated receptors

PPARs are a family of nuclear receptors which act as lipid activated transcription factors. This family consists of three different isoforms: PPARα, PPARβ/δ, and PPARγ. These three receptors have different tissue distributions and distinct biological roles. However, each can affect both positive and negative regulation of inflammatory and metabolic genes. PPARs are activated by both endogenous ligands and synthetic drugs. Endogenous agonists include unsaturated fatty acids, eicosanoids, prostaglandins, components of low density lipoproteins, and derivatives of linoleic acid. The most commonly used synthetic agonists for PPAR receptors include the fibrates, which bind PPARα the thiazolidinediones (TZDs), or glitazones, which bind PPARγ and the glitazars, which bind both.

Canonically, PPARs form heterodimers with retinoid X receptors (RXRs) and bind to peroxisome proliferator response elements (PPREs) located in the promoter region of target genes. When inactive, PPAR-RXR is bound to a corepressor complex. Ligand binding to PPARs induces a conformational change and the release of the corepressor complex for degradation. The activated heterodimer then recruits a coactivator complex which facilitates gene expression. In their capacity as metabolic regulators, PPARs modulate several vital cellular functions including adipocyte differentiation, fatty acid oxidation, and glucose metabolism.

Research in the last decade has outlined another important function of PPARs: the inhibition of inflammatory gene expression. A study published in *Nature* by Jiang et al. (1998) was the first to demonstrate that both natural and synthetic PPARγ agonists could block the production of proinflammatory cytokines, TNFα, IL-6, and IL-1β, in cultured monocytes. In the course of their study, the authors made the intriguing observation that the nature of the inflammatory agent used to induce cytokine expression in monocytes effected the outcome of the PPARγ agonist treatment. Specifically, 15d-PGJ_2_ and troglitazone inhibited TNFα expression in monocytes stimulated by okadaic acid or phorbol ester but not lipopolysaccharide (LPS).

In the same issue of *Nature*, Ricote et al. (1998) presented evidence that activated macrophages upregulate PPARγ. They further demonstrated that ligand bound PPARγ inhibits inflammatory gene expression through a process termed *transrepression* by targeting specific transcription factors including NF-κB, AP-1, and STAT. Transrepression is any mechanism by which a nuclear receptor, when bound to a ligand, can repress gene expression by interaction with transcription factors and regulatory proteins, not by direct interaction with specific DNA sequences. There are several forms of transrepression, including histone modification, block of RNA polymerase hyperphosphorylation, coactivator complex disruption, coactivator complex competition, inhibition of corepressor clearance, etc. (Pascual and Glass, 2006).

### PPAR functions in inflammation

**Figure 1:**
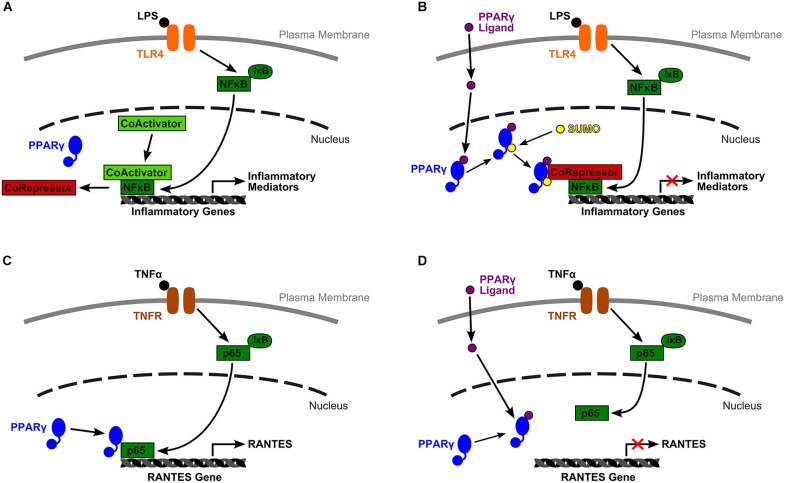
**Two models of PPARγ mediated inflammatory gene expression. (A)** Under basal conditions, inflammatory gene expression is inhibited by a corepressor complex. An inflammatory signal, such as lipopolysaccharide (LPS) binding to TLR4, initiates an inflammatory cascade. Inhibition of NF-κB by IκB is lifted, and NF-κB translocates to the nucleus. The corepressor complex is removed for degradation while NF-κB recruits a coactivator complex, binds to the target gene’s promoter, and initiates transcription. **(B)** Glass and colleagues (Pascual et al., 2005) proposed a mechanism by which activated PPARγ transrepresses inflammatory gene expression by inhibiting corepressor clearance. In their model, ligand binding to PPARγ allows receptor SUMOylation, which directs PPARγ to the NCoR-HDAC3 corepressor complex. PPARγ stabilizes this complex and prevents corepressor degradation, thus blocking gene transcription. **(C)** Wen et al. (2010) described a very different mechanism by which liganded and unliganded PPARγ have opposing effects on RANTES gene transcription. In their model, downstream TNFα inflammatory signals relieve NF-κB inhibition, phosphorylate the p65 subunit of NF-κB, and induce its nuclear translocation. There, unliganded PPARγ is *required* for successful association of p65 with the RANTES promoter. **(D)** However, ligand bound PPARγ is incapable of associating with p65, probably due to a conformational change, and RANTES expression is transrepressed.

While PPARα and β/δ have pertinent anti-inflammatory effects, the role of PPARγ as a negative regulator of inflammatory genes, has been more completely explored. As outlined above, inactivated PPARγ-RXR binds to a corepressor complex at PPREs preventing gene expression. However, according to Christopher Glass and colleagues (Pascual et al., 2005), PPARγ is also capable of transrepressing inflammatory gene expression in macrophages by inhibiting corepressor clearance (Figure 1). Under basal conditions, corepressor complexes suppress inflammatory gene expression. In an inflammatory state, signaling through receptors such as toll-like receptors (TLRs) begins an inflammatory cascade. First, repressor complexes are ubiquinated and degraded. Next, inhibition of NF-κB is relieved and it translocates to the nucleus where it binds to the promoter region of target genes, initiating transcription.

However, ligand binding to PPARγ allows receptor SUMOylation, and this event directs PPARγ to a specific nuclear corepressor/histone deacetylase 3 complex (NCoR-HDAC3) bound to inflammatory gene promoter regions. SUMOylated PPARγ stabilizes this complex and prevents its degradation by blocking the recruitment of ubiquinylation/19 s proteosome machinery that is typically responsible for corepressor complex removal prior to gene transcription. Activated PPARγ maintains the NCoR portion of the complex in place thus keeping the target gene inactive (Pascual et al., 2005). This research provides one mechanistic explanation for PPARγ’s change from gene activating to gene repressing.

Additional work by Wen et al. (2010) in mesangial cells of the kidney has outlined a separate mechanism by which unliganded and ligand bound PPARγ serve different functions in NF-κB pathway facilitated gene expression (Figure 1). They reported that PPARγ ligands, the natural agonist, 15d-PGJ_2_, and synthetic molecules, troglitazone and ciglitazone, were able to block TNFα induced, NF-κB dependent expression of RANTES (CCL5) and MCP-1 (CCL2). They specifically explored the mechanism by which suppression of RANTES was achieved. The authors reported that downstream signalers of TNFα binding relieve inhibition of the p65 subunit of NF-κB by IκB, then phosphorylate p65, and induce its translocation to the nucleus. Once there, p65 binds to unliganded PPARγ, a relationship that is required for p65 to bind to its target κB site at the RANTES promoter and facilitate gene transcription. Yet, when PPARγ binds a ligand, due probably to a conformational change, PPARγ can no longer associate with p65. Under these conditions, p65 is not able to bind to κB sites, thus RANTES expression is transrepressed (Wen et al., 2010). Again, this mechanism provides another method by which PPARγ can alter its actions from promoting gene expression to actively repressing transcription.

These two models demonstrate that transrepression is complex and achieved by various mechanisms that are situationally-specific. Only a small part of this process as it is played out in different cell types under different conditions has been illuminated. While PPAR agonists may hold great therapeutic potential, their actions are many and varied. Within their capability are many positive effects, but also undesirable side effects that have unfortunately limited their use. Uncovering the actions of these drugs sufficiently to separate their gene activating and gene repressing effects, inform more directed treatments, or even permit the development of “designer” pharmaceuticals whose side-effects are reduced will take significant further exploration (Glass and Saijo, 2010).

## PPAR agonists can alter chemokine expression

A large number of studies have investigated the effects of PPAR agonist administration on inflammatory mediator expression in many tissues and disease models. There is significant evidence from models of diabetes, arthritis, atherosclerosis, Parkinson’s disease, Alzheimer’s disease and others that administration of PPAR natural ligands and synthetic agonists has anti-inflammatory effects. Specific reductions in proinflammatory chemokines and cytokines has been observed in numerous cells types: renal cells (Wang et al., 2011; Lu et al., 2013), vascular smooth muscle cells (Marchesi et al., 2013), adipocytes (Guri et al., 2008; Ueno et al., 2012), mesothelial cells (Sauter et al., 2012), epithelial cells (Neri et al., 2011), splenocytes (Bassaganya-Riera et al., 2011), monocytes/macrophages (Han et al., 2005; Tanaka et al., 2005; Hounoki et al., 2008; Liu et al., 2012), astrocytes (Lee et al., 2008, 2012), and microglia (Kim et al., 2012).

### MCP-1/CCL2 expression

As discussed above, signaling between monocyte chemoattractant protein-1 (MCP-1) and its cognate receptor, CCR2, has garnered a great deal of attention by researchers seeking to identify those chemokines that play the most important roles in neuroinflammation and neuropathic pain. MCP-1/CCR2 signaling has demonstrated some non-redundant effects, particularly in monocyte/macrophage recruitment, which make these two a most promising therapeutic target. For example, Abbadie et al. (2003) showed that CCR2−/− mice show a pain free phenotype after sciatic nerve ligation, a model of neuropathic pain, and a marked decrease in nociceptive behavior after formalin injection, a model of inflammatory pain, when compared with controls. Further, MCP-1 and CCR2 remain upregulated for a long period after injury in several models. This evidence suggests that they serve a long-lasting function.

Information on PPARγ agonist induced inflammatory gene repression in nervous system cells types is limited. Real time PCR data on whole CNS tissue homogenate has shown suppression of MCP-1 expression by TZDs in an ischemic stroke model (Tureyen et al., 2007), a traumatic brain injury model (Yi et al., 2008), and a spinal cord injury model (Park et al., 2007). In the latter case, TZDs also conferred a number of neuroprotective effects (decreased lesion size, motor neuron loss, myelin loss, astrogliosis and microgliosis, and increased motor function recovery) via a PPARγ dependent mechanism.

An early study in Paul Drew’s lab (Kielian et al., 2004) tested the effects of 15d-PGJ_2_ effects on many cytokines and chemokines. In a model of brain bacterial infection, 15d-PGJ_2_ reduced microglial expression of several proinflammatory cytokines including MCP-1. The group followed up with a series of parallel studies (Storer et al., 2005a,b; Xu et al., 2005) that tested the efficacy of endogenous and synthetic PPAR ligands on proinflammatory cytokine and chemokine inhibition in LPS stimulated cultured microglia and astrocytes. Both prostaglandin PPARγ agonists, 15d-PGJ_2_ and PGA2, strongly inhibited MCP-1 production in microglia. Rosiglitazone also robustly decreased MCP-1 expression, but ciglitazone did so only at the highest tested doses, while pioglitazone had no effect. Astrocytes showed greater resistance to PPARγ agonist induced MCP-1 repression. PGA2 strongly inhibited MCP-1 upregulation while 15d-PGJ_2_ had a modest improving effect. However, all the TZDs had an effect only at the very highest dose. Finally, fibrates, synthetic PPARα agonists, also blocked MCP-1 expression in microglia.

Like astrocytes and microglia, resident and circulating immune cells also play a large role in neuropathic pain. PPARγ is upregulated in macrophages during inflammation, and agonists can reduce the inflammatory migration, proliferation, infiltration, and phagocytotic ability of these cells (Ito et al., 2003; Tureyen et al., 2007; Hounoki et al., 2008; Liu et al., 2012). MCP-1/CCR2 signaling in macrophages is a target for PPARγ agonists. Treated monocytes/macrophages show decreased migration toward MCP-1 (Kintscher et al., 2000; Tanaka et al., 2005) and reduced MCP-1 expression (Rival et al., 2002).

Researchers have also reported that activated PPARβ/δ can repress MCP-1 expression in macrophages (Lee et al., 2003; Tan et al., 2005). Lee et al. (2003) reported a mechanism by which ligand bound and unliganded PPARβ/δ achieves differential regulation of MCP-1 expression in macrophages, which strongly echoes the mechanism for PPARγ regulation of RANTES expression described by Wen et al. (2010), above. Lee et al. revealed that the presence of PPARβ/δ in macrophages was associated with proinflammatory effects which were; however, completely blocked by the introduction of a PPARβ/δ agonist, GW501516. They suggested that unliganded PPARβ/δ interacts with other transcription factors to promote expression of MCP-1 and other proinflammatory cytokines.

CCR2 is also a target for activated PPARγ research shows that the two promoters which control CCR2 expression in monocytes are both subject to repression by ligand bound PPARγ (Chen et al., 2005). PPARγ agonists decrease infiltration by CCR2+ monocytes (Guri et al., 2008) likely by blocking CCR2 gene transcription (Tanaka et al., 2005). In one study, simvastatin, from the statin family of drugs used commonly for atherosclerosis management, was able to activate a peroxisome-proliferator response element in a PPARγ dependent manner to produce effects similar to those achieved by PPARγ agonists. Simvastatin treated monocytes failed to migrate toward MCP-1 probably because they had significantly decreased levels of CCR2 mRNA and protein (Han et al., 2005).

### RANTES/CCL5 expression

RANTES (regulated on activation, normal T cell expressed and secreted; CCL5) is another chemokine with a demonstrated role in pain behavior and sensitization. RANTES binds the CCR5 chemokine receptor which is known as an HIV-1 coreceptor. RANTES serves as a chemoattractant for memory T helper cells and leukocytes including blood monocytes and eosinophils. CCR5 expression on primary sensory neurons (Oh et al., 2001) has been demonstrated. RANTES delivery both in the periphery (Conti et al., 1998; Oh et al., 2001) and the central nervous system (Benamar et al., 2008) causes pain hypersensitivity. Finally, RANTES−/− mice show decreased nociceptive sensitivity and reduced macrophage recruitment after peripheral nerve injury (Liou et al., 2012). While more remains to be determined about the specific mechanisms by which RANTES participates in neuropathic pain, this chemokine clearly plays a role in peripheral sensitization.

In the case of RANTES, even less information exists than does for MCP-1 regarding the ability of PPAR agonists to alter its expression in nervous system cells. Only one such study has connected changes in PPAR signaling with a decrease in RANTES expression. Xiao et al. (2010) studied the effects of steroid receptor coactivator-3 (SRC-3) deficiency in experimental autoimmune encephalomyelitis (EAE) induced mice. SCR-3 is a p160 family coactivator that can transactivate nuclear receptors, including PPARs. They reported that SRC3−/− mice showed decreased disease severity and correlated a decrease in chemokine (RANTES, MCP-1, MIP-1α, and IP-10) expression with an increase in PPARβ/δ expression. The authors hypothesized that increased PPARβ/δ signaling altered the activation state of resident microglia, promoting an anti-inflammatory profile, as evidenced by an increase in IL-10 and other anti-inflammatory mediators (Xiao et al., 2010).

PPARγ agonists reduce RANTES expression in some immune cells as well. PPARγ activation blocks RANTES expression in immature dendritic cells (Szanto and Nagy, 2008). Interestingly, while prostaglandins reduce RANTES expression in LPS stimulated peritoneal macrophages, TZDs were unable to replicate this effect (Kim and Kim, 2007). The authors determined that 15d-PGJ_2_ and PGA were acting via a PPARγ independent mechanism. While 15d-PGJ_2_ altered RANTES expression in differentiated macrophages, it had no effect on either mRNA or protein levels of RANTES in peripheral blood monocytes, indicating that differences in cell maturity constitute another situationally-specific outcome of drug administration.

RANTES is expressed in many other tissue types during inflammatory diseases. Animal models of inflammation in lung (Arnold and König, 2006), gastric (Cha et al., 2011), and renal (Li et al., 2005; Zhang et al., 2006; Wen et al., 2010) tissues show that PPARα and γ activation can reduce RANTES levels. As outlined above, Wen et al. (2010) described another transrepression mechanism by which liganded and unliganded PPARγ have opposing effects on RANTES expression through different interactions with the p65 subunit of NF-κB. Lastly, in human endometrial stromal cells, Pritts et al. (2002) demonstrated that rosiglitazone and 15d-PGJ_2_ act at an upstream PPRE on the RANTES promoter to decrease the chemokine’s transcription, showing that canonical PPARγ behavior may also have anti-inflammatory results.

### MIP-1α/CCL3

MIP-1α (macrophage inflammatory protein-1α CCL3) is strongly upregulated throughout the pain neuraxis after nervous system injury. Increase in MIP-1α expression has been reported locally in Schwann cells and infiltrating macrophages after sciatic nerve injury (Kiguchi et al., 2010b) as well as in macrophages in the dorsal root ganglion (Kim et al., 2011). Both peripheral (Kiguchi et al., 2010a) and central (Knerlich-Lukoschus et al., 2011b) nervous system injuries cause upregulation of MIP-1α and it’s receptor, CCR1, in the spinal cord. Traumatic spinal cord injury also increases the expression of MIP-1α and MCP-1 in the thalamus, hippocampus, and periaquaductal gray (Knerlich-Lukoschus et al., 2011a). Chemokine levels stay elevated for weeks after injury and MIP-1α/CCR1 expression correlates well with nociceptive behavior (Knerlich-Lukoschus et al., 2011b).

There is minimal data in the literature examining PPAR agonist modulation of MIP-1α expression in the nervous system. In one example of neuropathy, bacterial brain abscess, ciglitazone had neuroprotective and anti-inflammatory effects. Ciglitazone treatment decreased microgliosis overall, but increased phagocytotic activity by microglia. Additionally, protein levels of MIP-1α as well as other proinflammatory mediators (TNFα, IL-1β, and CXCL2) were decreased in the abscessed tissue (Kielian et al., 2004).

PPARγ signaling is also linked to decreased proinflammatory cytokine and chemokine expression in immune cells elsewhere in the body. Malur et al. (2009) demonstrated the importance of PPARγ expression in alveolar macrophages to maintain lung homeostasis. The authors reported that deletion of PPARγ in alveolar macrophages promoted a Th1 type inflammatory response including an upregulation of MIP-1α and IP-10. They proposed the use of PPARγ agonists for inflammatory lung diseases. However, an earlier study reported that 15d-PGJ_2_ treatment enhanced lung inflammation caused by LPS in a mouse model. Instead of producing an anti-inflammatory response, 15d-PGJ_2_ increased edema as well as proinflammatory chemokine (MIP-1α and MCP-1) and cytokine (IL-1β) expression.

A related study by Gosset et al. (2001) in mature dendritic cells showed that PPARγ activation yielded variable effects on chemokine expression depending upon the inflammatory agent employed. In once case, stimulation by a CD40 ligand, TZDs decreased the induced expression of MIP-1α as well as RANTES and IP-10. However, when LPS was used, TZDs had no effect on MIP-1α expression. This work, like that by Gurley et al. (2008) discussed below, demonstrates the situationally-specific nature of cellular responses to PPAR agonists.

### Fractalkine/CX3CL1

Fractalkine, also designated CX3CL1 for the three amino acids that separate the characteristic N-terminal cysteines, is a unique chemokine. It is the only chemokine that can remain adhered to cells by means of a mucin-like stalk that tethers the chemokine domain to the plasma membrane. Cleavage by cathepsin S releases a soluble form of fractalkine (Clark et al., 2009). Fractalkine binds to CX3CR1, the fractalkine receptor, and is chemoattractive for T-cells and monocytes. Endothelial cells express the tethered form of fractalkine during inflammation. Its unique structure allows fractalkine to attract circulating leukocytes and assist in adhering them to the endothelium.

In chronic pain states, studies have shown a key role for fractalkine and the fractalkine receptor in microglial activation (Verge et al., 2004; Lindia et al., 2005; Yang et al., 2012). The fractalkine receptor is primarily expressed in microglia in pain related areas of the dorsal horn (Lindia et al., 2005). Intrathecal delivery of soluable fractalkine produces nociceptive behavior in animal models (Milligan et al., 2004; Zhuang et al., 2007). CX3CR1−/− mice show decreased neuropathic pain and microglial activation (Staniland et al., 2010).

In spite of abundant information about the role of fractalkine and its receptor in neuropathic pain, no studies have yet demonstrated the ability of any PPAR agonist to alter their expression in the nervous system. However, PPARγ activation has demonstrated ability to reduce fractalkine expression by inflamed endothelial cells as well as decreased fractalkine receptor expression on monocytes/macrophages (Imaizumi et al., 2002; Bursill et al., 2010; Wan and Evans, 2010). Barlic and Murphy (2007) reported that this PPARγ activation regulates a change in CCR2^hi^/CX3CR1^low^ monocytes promoting a change to CCR2^low^/CX3CR1^hi^ macrophages. Finally, Wan and Evans (2010) in their paper showing negative regulation of fractalkine receptor expression by rosiglitazone also demonstrated that an agonist to PPARβ/δ decreased fractalkine receptor expression albeit to a lesser extent than rosiglitazone.

Interestingly, there is evidence that fractalkine signaling may modulate PPARγ receptor expression. Mizutani et al. (2007) revealed that low levels of fractalkine/fractalkine receptor signaling promotes an increase in PPARγ expression, thus maintaining a low level of anti-inflammatory activity in intestinal macrophages. They point out that intestinal macrophages are, by necessity, hyporeactive to inflammatory stimuli. Similar to the relationship between PPARγ and MIP-1α in alveolar macrophages (Malur et al., 2009), these authors hypothesize that very low levels of fractalkine signaling help maintain intestinal homeostasis by modulating PPARγ expression.

### SDF-1/CXCL12

SDF-1 (stromal cell derived factor-1; CXCL12) is an evolutionarily old chemokine that serves key functions in stem cell migration and organ development for example in hematopoiesis, angiogenesis, and neurogenesis, as well as playing a part in inflammation. Along with other chemokines, peripheral administration of SDF-1 is pronociceptive (Oh et al., 2001). The SDF-1 receptor, CXCR4, is expressed in dorsal root ganglion neurons, and its expression is upregulated after peripheral nerve injury (Oh et al., 2001; Bhangoo et al., 2007). SDF-1 and CXCR4 expression is also upregulated in the spinal cord in a model of traumatic spinal cord injury (Knerlich-Lukoschus et al., 2011b). SDF-1/CXCR4 signaling has been implicated in HIV-1 associated pain; CXCR4 is a known HIV-1 coreceptor like CCR5 (Bhangoo et al., 2009). Finally, SDF-1/CXCR4 may also involved in mediating opioid induced neuropathic pain (Wilson et al., 2011).

A small body of evidence indicates that activated PPARγ signaling can block SDF-1/CXCR4 facilitated lymphocyte chemotaxis as well as decrease both chemokine and receptor expression. Walcher et al. (2008) demonstrated that PPARγ activation can, within minutes, reduce SDF-1 induced migration of CD4+ lymphocytes (Walcher et al., 2008). This suggests some immediate interference with an SDF-1 receptor, rather than any change in gene expression. However, PPARγ agonists have been shown to reduce SDF-1 expression in adipose tissue (Foryst-Ludwig et al., 2010) and aortic grafts (Onuta et al., 2007), both inflammatory disease models. Natural ligands and TZDs have reduced CXCR4 expression in tumor cells in a model of metastasizing cancer (Richard and Blay, 2008). The authors cited disruption of SDF-1/CXCR4 signaling in the metastasis of stem-like cancer cells by a PPARγ dependent mechanism as a possible new cancer control treatment.

## PPARγ agonist actions may be receptor dependent or receptor independent

**Figure 2:**
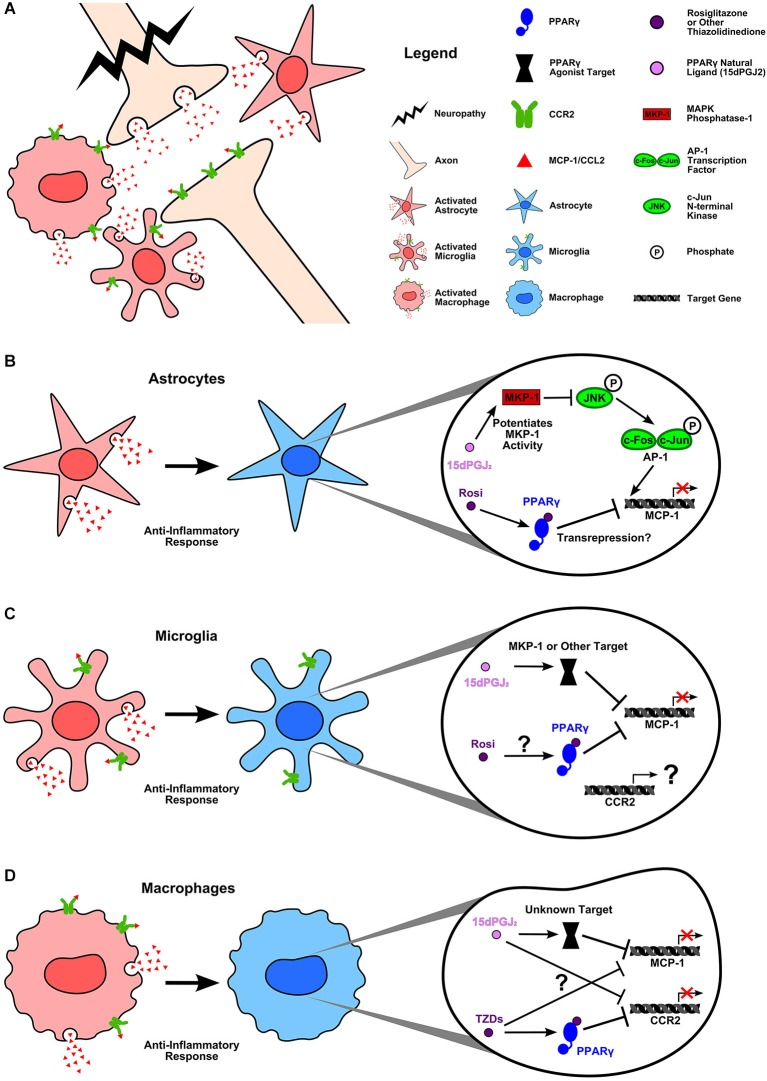
**PPARγ agonists inhibit MCP-1 and CCR2 expression in inflammatory neuropathy**. **(A)** Damage to the central nervous system causes activation of astrocytes and resident microglia as well as recruited macrophages. Glial cells (Van Der Voorn et al., 1999; Abbadie et al., 2003; Yan et al., 2007; Zhang et al., 2007; Zhang et al., 2012; Knerlich-Lukoschus et al., 2008) and macrophages as well as neurons (Zhang and De Koninck, 2006; Gao and Ji, 2010; Zhang et al., 2012) upregulate MCP-1 and CCR2 expression as part of the inflammatory response to injury. **(B)** Activated astrocytes express MCP-1, which can be blocked by rosiglitazone and 15d-PGJ_2_. Lee et al. (2008) demonstrated that 15d-PGJ_2_ inhibits INF-γ induced MCP-1 expression by potentiating the activity of MAPK phosphatase-1. MKP-1 targets JNK for dephosphorylation. This prevents the activation of the AP-1 transcription factor subunit, c-jun, thus inhibiting AP-1 mediated MCP-1 expression. In the case of rosiglitazone, it is unclear what mechanism is used to block MCP-1 expression; however, Lee et al. (2008) confirmed that rosiglitazone acts via PPARγ to inhibit INF-γ induced MCP-1. **(C)** Activated microglia upregulate MCP-1 and CCR2 during inflammation. Again, both rosiglitazone and 15d-PGJ_2_ can block MCP-1 expression. While rosiglitazone’s mechanism of action remains unclear, studies have verified that 15d-PGJ_2_ is acting in a PPARγ independent manner (Lee et al., 2008; Kim et al., 2012). Lee et al. (2008) reported that, as in astrocytes, 15d-PGJ_2_ acts upon MKP-1 to block INF-γ induced MCP-1 expression in microglia. No studies have yet examined the effects of natural or synthetic PPARγ agonists on CCR2 expression in activated microglia. **(D)** Recruited macrophages express both MCP-1 and CCR2. Thiazolidinediones (TZDs) decrease monocyte migration toward MCP-1 (Kintscher et al., 2000; Tanaka et al., 2005) likely by PPARγ dependent inhibition of CCR2 gene expression (Chen et al., 2005). However, whether or not TZDs act in a PPARγ dependent manner to block MCP-1 expression is unknown (Hounoki et al., 2008). In the case of 15d-PGJ_2_, studies again indicate a PPARγ independent mechanism of action for decreasing LPS induced MCP-1 expression (Liu et al., 2012). 15d-PGJ_2_ has a demonstrated ability to decrease CCR2 mRNA, yet the mechanistic target remains to be discovered (Tanaka et al., 2005). The ability of PPARγ agonists to decrease MCP-1 and CCR2 expression in cell types known to be involved in neuroinflammation and pain is encouraging. PPARγ agonists offer tantalizing hope of blocking proinflammatory chemokine signaling between glial cells, immune cells, and neurons which is known to be fundamental to neuropathic pain. However, these drugs have many and varied targets which complicates their use at present. Further research is needed to identify the mechanisms by which both natural and synthetic PPAR agonists reduce inflammation in the nervous system. Such knowledge will help researchers to identify the agonists best suited to preventing chronic inflammatory chemokine expression.

Although PPARγ agonists have proven able to reduce inflammatory gene expression, to what degree these agents require the PPARγ receptor to mediate their effects is still unclear. The evidence indicates that it is common for endogenous PPARγ ligands, particularly 15d-PGJ_2_, to exert effects via PPARγ independent mechanisms. For example, Lee et al. (2008) demonstrated that when 15d-PGJ_2_ decreases MCP-1 expression in INF-γ stimulated astrocytes it does so not by binding PPARγ but instead by modulating MAPK-phosphatase 1 (Figure 2). Many other studies have confirmed that at least some of the anti-inflammatory actions of 15d-PGJ_2_ are PPARγ independent (Hounoki et al., 2008; Kim et al., 2012; Liu et al., 2012).

However, it is not only 15d-PGJ_2_ that shows PPARγ independent activity. Welch et al. (2003) published data revealing that rosiglitazone utilizes two different mechanisms, depending upon its concentration, to alter proinflammatory gene expression in macrophages. Rosiglitazone inhibits production of LPS and INF-γ target genes via a PPARγ dependent mechanism at low doses, but at high doses it employs a PPARγ independent mechanism. The authors noted that the inhibition dose-response curve for rosiglitazone did not match its established binding affinity for PPARγ. So, using PPARγ−/− macrophages, they demonstrated that rosiglitazone still repressed proinflammatory genes and determined that rosiglitazone was binding to PPARβ/δ.

Finally, there is evidence that the effects of different PPARγ agonists may be a function of additional, modulatory signals. Gurley et al. (2008) demonstrated that pioglitazone and troglitazone could have varying effects in activated astrocytes depending upon the nature of a coadministered TLR ligand. They reported no change in MCP-1 expression after LPS (TLR4 ligand) and troglitazone. The same was true of single stranded RNA (TLR7/8 ligand) with troglitazone; yet ssRNA and pioglitazone facilitated an increase in MCP-1 expression. Most fascinating, when flagellin (TLR5 ligand) and pioglitazone were given, MCP-1 expression increased; however, when flagellin was accompanied by troglitazone, MCP-1 expression decreased.

From these data, we can gather that PPARγ agonist modes of action are complex, as are the variety of ways in which liganded PPARγ can facilitate either gene expression or transrepression. Further modification of activated PPARγ actions by other ligand-receptors and their intracellular signals, can also yield different results. Significant work remains to be done to elucidate such situationally-specific mechanisms in order to determine why some treatments work and others fail.

## PPAR agonists modulate neuropathic pain

As noted earlier, the use of PPAR agonists as a treatment has been explored in animal models of inflammation, brain injury, demyelination, and pain. The results of many of these studies are encouraging. PPAR agonists have been shown, in animal neuropathy models, to possess neuroprotective (decreased lesion volume), anti-inflammatory (decreased microglial activation and inflammatory gene expression), antiapoptotic (decreased number of apoptotic neurons), antioxidative, and neurologically improving effects (Drew et al., 2005; Zhao et al., 2005; Racke et al., 2006; Park et al., 2007; Costa et al., 2008; Yi et al., 2008; Di Cesare Mannelli et al., 2013). As the inflammation following neuropathy is strongly linked to the development of neuropathic pain states, it is reasonable to ask whether or not PPAR agonists can modulate neuropathic pain behavior in a manner similar to their anti-inflammatory effects.

### Use in humans

Evidence from several clinical trials demonstrates that the endogenous PPARα agonist, palmitoylethanolamide (PEA), is an effective treatment for various human pain conditions. PEA was identified in 1957 as a fatty acid amide with anti-inflammatory properties (Kuehl et al., 1957). While PEA is a known agonist of PPARα, its anti-inflammatory effects may be mediated by additional receptors, including the other PPAR isoforms as well as TRPV1 and cannabinoid receptors. Further, PEA appears to have many possible target cells. Additional research is needed to expand our understanding of the mechanisms that underlie PEA’s effects.

PEA is available in some European countries as a dietary supplement for medical purposes under the names Normast^®^ and PeaPure^®^ indicated for the treatment of pain and inflammation. It has demonstrated great efficacy in treating neuropathic pain, even in patients whose pain has proven refractory to other therapies (Biasiotta et al., 2010). Clinical trials have been conducted in patients with diabetic neuropathy (Schifilliti et al., 2014), postoperative pain, sciatic pain, multiple sclerosis pain (Kopsky and Keppel Hesselink, 2012), chemotherapy pain (Truini et al., 2011), and post-stroke pain, among other conditions (Keppel Hesselink (2012) published a detailed review of studies using PEA to treat chronic pain).

Several characteristics of PEA make it a very attractive pain therapy. The first, mentioned above, is that it has been successful at reducing pain in patients whose conditions were either unaffected or incompletely treated by other medications. Second, both clinical trials and case studies have reported no side effects of PEA use. The lack of side effects has encouraged physicians to include PEA alongside more traditional pain medications such as oxycodone and pregabalin in a multimodal treatment plan. PEA has shown no drug-drug interactions when given with these medications. In fact, in several studies the addition of PEA to an existing treatment regimen has increased the therapeutic effectiveness and in some cases permitted a dose decrease of companion drugs. PEA has also been successful in combination with non-drug treatments such as physical therapy and acupuncture (Desio, 2010; Keppel Hesselink, 2012; Keppel Hesselink and Hekker, 2012; Kopsky and Keppel Hesselink, 2012; Schifilliti et al., 2014; Skaper et al., 2014).

Most recently, Sasso et al. (2013) published a study regarding a novel method for manipulating the anti-inflammatory and antinociceptive effects of PEA-PPARα signaling in animal models. These authors reported on a novel N-acylethanolamine acid amidase (NAAA) inhibitor, ARN077, which indirectly prevents the degradation of PEA. PEA is produced endogenously from precursors (fatty acid ethanolamides) by N-acyl-phosphatidylethanolamide phospholipase D as needed, and its levels are controlled by NAAA mediated hydrolysis. Sasso et al. reported that ARN077 attenuated neuropathic pain behavior by inhibiting NAAA activity and preserving PEA levels. Thus, maintaining PEA levels in injured tissues either by addition of exogenous PEA or preservation of endogenous PEA appears to be an effective pain treatment (Taylor, 2013). Indeed, if ARN077 were to prove an effective therapy in humans, it might serve well given in conjunction with Normast^®^ or PeaPure^®^.

### A note on Thiazolidinediones

There is very little information regarding the use PPARγ agonists for neuropathic pain treatment in humans. In part, this is the result of conflicting data about the safety of key agonist, rosiglitazone. In 2007, Nissen and Wolski, published a meta-analysis of the cardiovascular side effects of rosiglitazone (Avandia^®^) treatment for type II diabetes mellitus. They concluded that rosiglitazone use was associated with an increased risk of myocardial infarction. In spite of a rebuttal publication by the RECORD (Rosiglitazone Evaluated for Cardiac Outcomes and Regulation of Glycaemia in Diabetes) study group (Home et al., 2007), the United States Food and Drug Administration (FDA) in 2010 imposed strong restrictions on rosiglitazone use in patients.

On November 25, 2013, the FDA delivered a press release announcing the removal of the majority of these restrictions on the prescription and use of Avandia after the final results of the RECORD clinical trial [NCT00379769] (Home et al., 2009) failed to uphold the findings of Nissen and Wolski.^1^ The RECORD study results are a welcome development for rosiglitazone and other thiazolidinedione drugs which have shown such promise for treating diabetes and other conditions.

### In animal models

Animal research has provided evidence that both natural and synthetic ligands to PPARα and PPARγ reduce pain. Agonists with demonstrated pain alleviating effects include the aforementioned rosiglitazone, pioglitazone, and 15d-PGJ_2_ as well as PEA and fenofibrate. Other synthetic PPARα agonists, GW7647 and Wy14643, also reduce pain. While these results are very encouraging, there remains a major challenge in assessing the collective results of animal experiments. The wide variety of pain models, drugs, drug doses and schedules, drug administration routes, pain assessment methods, pain assessment timepoints, and limited investigation into the method(s) of drug action make the identification of unifying themes extremely difficult. However, some general conclusions can be drawn. The evidence indicates that *PPAR agonists modulate neuropathic pain in animal models…*

#### …by acting at targets throughout the pain neuraxis

The most potent PPAR agonist therapy requires repeated drug administrations beginning in the early phases of pain generation. It is logical that treatment will be more efficacious *before* the long-term changes underlying sensitization have been established. Yet, as dicussed above, PEA appears able to reduce even persistent pain in some clinical studies. Second, there is some confusion about the *in vivo* cellular targets of PPAR agonists. In some cases, different groups have published contradictory reports. Nevertheless, there is evidence that PPAR agonists can act to reduce pain at targets in the brain (D’Agostino et al., 2009; Morgenweck et al., 2010), in the spinal cord (Churi et al., 2008; Morgenweck et al., 2013), in the peripheral nervous system (LoVerme et al., 2006; Takahashi et al., 2011), and in the tissue (Hasegawa-Moriyama et al., 2012).

#### …primarily via PPAR dependent mechanisms

Wherever the location and cellular target(s) of PPAR agonists may be, the evidence points to PPARs as the primary mediators of pain alleviation by these agonists. In neuropathic pain models, researchers show that rosiglitazone (Park et al., 2007; Churi et al., 2008), pioglitazone (Park et al., 2007; Maeda et al., 2008; Jia et al., 2013; Morgenweck et al., 2013), and 15d-PGJ_2_ (Churi et al., 2008) all act via PPARγ and PEA acts via PPARα (LoVerme et al., 2006; Di Cesare Mannelli et al., 2013). The same is true in models of inflammatory pain (D’Agostino et al., 2009) as well as of the neuroprotective effects (Park et al., 2007; Genovese et al., 2008) observed with these agents.

Yet, as dicussed earlier, PPAR agonists very clearly have receptor independent effects. Although pain studies have repeatedly verified the PPARγ dependent actions of rosiglitazone, it has been shown that, at high enough concentrations, rosiglitazone associates with PPARβ/δ (Welch et al., 2003). In another case, researchers used antagonists to PPARγ and PPARβ/δ to show that PEA, although not an agonist for either receptor, nevertheless appears to exert some downstream effect via these receptors (Paterniti et al., 2013). Others have tested the contribution of PPARγ and PPARβ/δ to the antinociceptive effects of PEA and found no association (LoVerme et al., 2006), thus further research is needed to definitively address these conflicting reports. Similarly, Costa et al. (2008) published their findings that PEA utilizes *not* PPARα, but instead interacts with cannabinoid receptor type 1 (CB_1_), the transient receptor potential cation channel vanilloid receptor 1 (TRPV1), and PPARγ to reduce pain. Again, these results contradict the findings of other studies as mentioned above.

#### …producing both changes in gene transcription and non-transcriptional effects

Although the receptors involved in mediating the effects of PPAR agonists require further investigation, one downstream target of PPAR agonist signaling, NF-κB, has been clearly identified. Significant evidence shows that the results of PPAR agonist administration include block of IκB degradation, decreased p65 subunit phosphorylation, and a decrease in NF-κB translocation to the nucleus; the end result being a reduction in inflammatory gene expression (Dehmer et al., 2004; D’Agostino et al., 2007, 2009; Genovese et al., 2008).

However, research indicates that PPAR agonists have effects beyond those exerted upon transcription factors like NF-κB. Evidence shows that PPAR agonists, particularly rosiglitazone and PEA, can relieve pain rapidly but transiently (minutes-hours) (LoVerme et al., 2006; Churi et al., 2008; D’Agostino et al., 2009; Khasabova et al., 2012) as well as over the long-term (days) (Costa et al., 2008; Maeda et al., 2008; Jain et al., 2009; Takahashi et al., 2011; Jia et al., 2013). Thus, it seems clear that, in addition to effects that lead to modifications in gene transcription, these agonists must also have non-transcriptional targets. For example, LoVerme et al. (2006) reported that PEA administration resulted in a rapid decrease in the elecrophysiological response of spinal nociceptors to peripheral formalin injection.

#### …ultimately altering the expression of inflammatory mediators including chemokines and their receptors

While the mechanistic underpinnings PPAR agonist actions are known to be many and varied, the impact of these agents inhibitors of inflammation is well supported. Indeed, many studies have shown that PPAR agonists decrease the levels of upstream inflammatory cytokines known to induce chemokine expression, including TNFα, IL-1β, and IL-6 (Storer et al., 2005a,b; Park et al., 2007; Loría et al., 2008; Maeda et al., 2008; Impellizzeri et al., 2013; Jia et al., 2013; Paterniti et al., 2013).

In a few cases, specific decreases in chemokine expression have been reported in studies examining the effects of PPAR agonists on animal pain conditions. Impellizzeri et al. (2013) reported decreases in MIP-1α and MIP-2 levels after treatment with PEA and luteolin (an antioxidant) in a mouse model of rheumatoid arthritis. Park et al. (2007) demonstrated that pioglitazone decreased MCP-1 expression in spinal cord tissue in a model of traumatic spinal cord injury. Finally, Takahashi et al. (2011) observed a decrease in CCR2 expression in rosiglitazone-treated macrophages. In their study, the authors were able to achieve pain relief by transplanting these treated macrophages directly at the site of partial sciatic nerve ligation. It is possible that this result is part of a greater rosiglitazone effect on macrophages, as treatment with this drug seems to promote a polarity change from M1 (pro-inflammatory) to M2 (anti-inflammatory) (Hasegawa-Moriyama et al., 2012, 2013).

## Conclusions

In the 15 years since the first reports that PPARγ serves functions in inflammation as well as metabolic regulation, researchers have opened the door on a subject of breathtaking complexity. In even these, earliest studies, investigators had begun to identify important questions about PPAR agonist actions that remain highly relevant today (Jiang et al., 1998; Ricote et al., 1998; Spiegelman, 1998).

The literature on PPAR signaling provides ample evidence that PPAR agonist administration can produce situationally-specific effects. These effects are the result, at least in part, of the ability of PPAR agonists to harness receptors other than PPARs, and to interact not only with transcription factors to impact gene expression but also to act at non-transcriptional targets to produce more rapid effects. To complicate matters further, the nature of those “situations” which generate different effects are not fully understood. In some cases, PPAR agonists known to bind to the same PPAR isoform, when administered under identical conditions can yield different results. Gurley et al. (2008) demonstrated this by showing that pioglitazone and troglitazone, both synthetic PPARγ agonists, produced opposite effects on flagellin induced MCP-1 expression. In other cases, agonists with the ability to act at the same PPAR isoform, achieve an identical effect by completely different mechanisms. For example, Lee et al. (2008) reported that rosiglitazone acted via a PPARγ dependent mechanism to decrease MCP-1 expression, while 15d-PGJ_2_, which is a natural ligand for PPARγ nevertheless employed a PPARγ independent mechanism (MAPK signaling) to achieve the same result.

Research in animal models shows that disrupting the signaling of important inflammatory chemokines is sufficient to achieve pain relief. Yet, the results of efforts to translate these findings to effective pharmaceuticals have been disappointing. It has been speculated that redundancy in chemokine signaling prevents a specific chemokine receptor antagonist, for example, from proving clinically effective. The heterogeneous nature of neuropathic pain also presents a worrying medical problem. PPAR agonists have a demonstrated ability to alter the expression of chemokines, their receptors, and the upstream inflammatory cytokines typically responsible for stimulating chemokine expression. While, these broad-spectrum effects are potentially the key to the ability of PPAR agonists to reduce pain, they have also yielded some problematic side effects.

### Future directions

Given this prohibitive complexity, the question arises: why is it valuable to pursue greater understanding of PPAR agonists? There are two important reasons. The first is that these agents, both natural and synthetic, are extremely powerful. Continued investigation into how PPAR agonists achieve anti-inflammatory and antinociceptive effects is vital. Unlocking these mechanisms of action has the potential to inform new, safer, and more effective therapies. Second, these agonists are already being used effectively in clinical settings. Whether it be PeaPure^®^ for pain management or Avandia^®^ for insulin sensitization, PPAR agonists have clear, medical value which might yet be expanded if clinical trials using these agonists to treat conditions from cancer to dementia prove fruitful. PEA in particular has shown unprecedented potential to treat neuropathic pain. The apparent absence of side effects and drug interactions is very promising. Further, researchers and clinicians ought not overlook a treatment that has, even occasionally, proven effective where other therapies failed.

As stated earlier, Spiegelman (1998) identified two important questions raised by the works of Jiang et al. and Ricote et al. which remain relevant today. First, what underlies the situationally-specific outcomes of PPAR agonist treatment? For example, why do PPARγ agonists yield different results depending upon the particulars of the inflammatory response? Second, what are the targets acted upon by PPAR ligands when PPAR independent effects are seen? What are the relative contributions of PPARs vs. other targets to the various results of PPAR agonist treatment?

Concerning the particular effects of PPAR agonists on chemokine expression, there are additional questions and directions. First, PPAR agonists have a demonstrated ability to effect the expression of chemokines. More evidence is needed from pain models reporting the results of PPAR agonist treatment on chemokine expression in the nervous system in areas and cell types where chemokine signaling is known to contribute to pain. All PPAR isoforms are known to be expressed to some extent in parts of the central and peripheral nervous systems, although the literature has shown that their presence may not be required for some agonists to effect chemokine expression (Moreno et al., 2004; van Neerven and Mey, 2007; Maeda et al., 2008; Wang et al., 2012).

An additional question is: to what degree do PPAR agonists alter chemokine expression directly vs. altering the expression of upstream, inflammatory cytokines? There is abundant data demonstrating that PPAR agonists decrease the levels of cytokines such as TNFα, IL-1β, and IL-6 amongst others. This effect alone might be responsible for a concomitant decrease in chemokine expression. Yet, there is also evidence for direct action of ligand bound PPARs at chemokine promoters and other regulatory sites. Activated PPARs appear able to target RANTES expression both via “canonical” behavior and transrepression (Pritts et al., 2002; Wen et al., 2010). There is evidence for differential regulation of MCP-1 by activated PPARβ/δ (Lee et al., 2003). Finally, the promoters for CCR2, the receptor for MCP-1, are targets for activated PPARγ (Chen et al., 2005).

In conclusion, PPAR agonists are powerful agents with wide-ranging anti-inflammatory effects. Studies in animal models show these compounds have potent antinociceptive effects as well. Indeed, the PPARα agonist, PEA, has made a promising start as a treatment for human neuropathic pain conditions. Much work remains to be done to understand the complex mechanisms by which PPAR agonists achieve their anti-inflammatory and antinociceptive effects. However, the evidence to date shows that PPAR agonists reduce the expression of many inflammatory mediators, including specific chemokines that are known to generate and maintain chronic pain. We believe that PPAR agonists represent an exciting new way to manage chemokine expression in situations of neuroinflammation and pain.

## Conflict of interest statement

The authors declare that the research was conducted in the absence of any commercial or financial relationships that could be construed as a potential conflict of interest.

## Footnotes

^1^www.fda.gov/NewsEvents/Newsroom/PressAnnouncements/ucm376516.htm
